# Outcome after surgical repair of partial anomalous pulmonary venous connection

**DOI:** 10.12669/pjms.326.10961

**Published:** 2016

**Authors:** Tariq Waqar, Zubair Ahmad Ansari, Mirza Ahmad Raza Baig

**Affiliations:** 1 Tariq Waqar, MBBS, FCPS, FRCS. Associate Professor of Pediatric Cardiac Surgery, CPE Institute of Cardiology, Multan, Pakistan; 2 Zubair Ahmad Ansari, FCPS. Senior Registrar Cardiac Surgery, CPE Institute of Cardiology, Multan, Pakistan; 3 Mirza Ahmad Raza Baig, BSc. Hons. CPT. Clinical Perfusionist, CPE Institute of Cardiology, Multan, Pakistan

**Keywords:** Partial anomalous pulmonary venous connection (PAPVC), Superior vena cava (SVC), Atrial septal defect (ASD), Intra cardiac baffle

## Abstract

**Objective:**

To review the operative results of 55 cases of partial anomalous pulmonary venous connection (PAPVC).

**Methods:**

This retrospective case series of 55 cases of PAPVC operated from January 2011 to June 2016 at CPE Institute of cardiology, Multan. Baseline characteristics of patients, their operative findings and results were retrieved from the hospital record.

**Results:**

Operation for PAPVC was performed in 55 patients. Patient’s age varied from 3-28 years (mean 12.56±7.49), their weight was 9-62 kg mean (25.61±16.28). There were 41(74.5%) males and 14(25.5%) females. 49(89.0%) patients had right sided PAPVC associated with ASD moreover 3(5.4%) cases had right pulmonary vein draining into right atrium. While there was only one case having left sided PAPVC (1.8%) and two cases (3.6%) of bilateral PAPVC (4%). Reassuringly, there was not a single mortality. However, one patient developed junctional rhythum, which was successfully controlled on amiodarone. SVC obstruction having 6mmHg gradient was observed in one case however patient is doing well and is on follow up since 9 months.

**Conclusion:**

Surgical correction of PAPVC generally carries highly reproducible results with low morbidity.

## INTRODUCTION

Partial anomalous pulmonary venous connection presents with variable anatomical patterns resulting in one or more pulmonary veins functionally draining into the right atrium or one of its venous tributaries instead of draining into left atrium.^1^ PAPVCs may occur as isolated anomalies or may be combined with ASD. The most common variant of PAPVC is the defect located in sinus venosus malformation i.e, superior caval atrial septal defect coexists with PAPVC.^2^,^3^ Other variants include right pulmonary vein draining into right atrium,^4^ connection of right pulmonary vein to IVC (scimitar syndrome) and rarely right pulmonary vein connects to azygos vein or coronary sinus.^5^,^6^ Similarly, left pulmonary vein may connect to left brachiocephalic vein through an anomalous vertical vein. Partial but bilateral PAPVC is rare.

Surgical correction of PAPVC varies according to the type of underlying anomaly, however the basic principle remains the segregtion of systemic and pulmonary circulation either by communicating the anomalous pulmonary vein to the left atrium directly or indirectly rerouting through baffle into left atrium.^7^,^8^ Our objective was to review the operative results of 55 cases of partial anomalous pulmonary venous connection (PAPVC).

## METHODS

Approval from ethical and administrative committee of CPE Institute of Cardiology, Multan was taken for research proposal of repair of partial anomalous pulmonary venous connection. Data was collected retrospectively from electronic hospital database at CPE Institute of cardiology, Multan. The 55 consecutive patients operated for partial anomalous pulmonary venous connection from January 2011 to June 2016 were included in this study.

### Operative Technique

Standard cardiopulmonary bypass was established in every patient using bicaval venus cannulation and ascending aortic cannulation. In patients with right-sided pulmonary veins draining at the junction of right atrium and superior vena cava (SVC), SVC was cannulated higher. But in those cases in which right pulmonary veins were draining into SVC, SVC was cannulated at the junction of SVC and innominate vein. In all cases, SVC was cannulated with right angled metal tip venous cannula. And for inferior vena cava cannulation, we used straight tip venus cannula. In all patients moderate hypothermia was achieved by lowering the body temperature to 30-32°C. Antegrade cold blood cardioplegia was used for cardiac arrest. For right-sided PAPVC to SVC, intra cardiac baffle with double patch technique was done in 39 patients in whom right superior pulmonary vein was draining into superior vena cava. Intracardiac baffle with single patch technique was done in 9 patients in whom right superior pulmonary vein was draining at the junction of right atrium and SVC. While warden procedure was done in one patient in which right superior pulmonary vein was draining into the upper part of SVC. For right sided PAPVC to right atrium, intra-atrial septum was excised to create atrial septal defect and pulmonary veins were rerouted into left atrium in two patients while in one patient there was secundum ASD already so pulmonary vein was rerouted by autologous pericardial patch after enlarging atrial septal defect. In left sided PAPVC, vertical vein that was draining left pulmonary veins to innominate vein was anastomosed side by side with left atrial appendage. Vertical vein was ligated close to innominate vein. For bilateral PAPVC, in one patient atrial septal defect was enlarged and coronary sinus was unroofed and single autologous patch was used to reroute the pulmonary veins and coronary sinus into left atrium. However, in second patient right pulmonary vein was rerouted by two pericardial patches and left pulmonary vein draining into vertical vein was rerouted by side to side anastomosis between vertical vein and left atrial appendage.

Milrinone and adrenaline was used in all patients as inotropic support for weaning from cardiopulmonary bypass, these inotropes were continued in intensive care unit. The patients in which there was pre-operative moderate to severe pulmonary hypertension, we electively kept these patients on ventilator for at least 24 hours and continued milrinone at the dose of 0.5 μg/kg/minute. To avoid pulmonary hypertension crisis we used prolonged ventilation with high FiO_2_, good sedation and pain management and anti-pulmonary hypertension medications like phosphodiesterase inhibitors (milrinone).

### Statistical Analysis

Following patient related search variables i.e. age, gender, weight, body surface area, disease diagnosis, surgical procedure performed, total cardiopulmonary bypass time, total cross clamp time, total number of blood transfusion, chest re-explorations, ICU stay (days), hospital stay (days), in-hospital mortality, operative mortality were searched from Cascade Database. Search results were generated in Microsoft Excel Sheet 2013. Any deficient or dubious data was reconfirmed from hospital paper record. Statistical information was analyzed by using Microsoft Excel 2013 and SPSS Version 16. Mean and standard deviation was utilized to report numeric variable and percentage was utilized to report proportions.

## RESULTS

Preoperative variables are shown in Table-I. Age range was from 3-28 years, mean age was 12.56±7.49. There were 41 (74.5%) male patients, while 14(25.5%) females. The weight ranged from 9 to 62 kg, mean weight was 25.61±16.28 kg. For right-sided PAPVC to SVC 49(89.0%), intracardiac baffle was done with double patch technique in 39 patients while 9 underwent intracardiac baffle with single patch technique and in one patient warden procedure was done. PAPVC to right atrium was repaired by single autologous pericardial patch to reroute anomalous vein. In left sided PAPVC, vertical vein was ligated at junction of innominate vein and proximal vertical vein was anastomosed with left atrial appendage.

**Table-I:** Preoperative baseline characteristic and types of PAPVC.

Variables		Values
Age (Y)		12.56±7.49
Gender (%)	Male	41 (74.5)
	Female	14 (25.5)
Weight (Kg)		25.61±16.28
Body Surface Area (kg/m^2^)		0.89±0.36
Types of PAPVD (%)		
Right Sided	Superior Vena Cave	49 (89.0%)
	Right Atrium	3 (5.4%)
Left Sided		1(1.8%)
Bilateral		2(3.6%)

PAPVD = Partial Anomalous Pulmonary Venous Drainage

Surgery was done under general anesthesia with standard cardiopulmonary bypass technique. Mean cardiopulmonary bypass time (CPB time) was 80.70±22.95 minutes and mean aortic cross clamp time was 45.46± 15.63 minutes. Ventilation mean time was 5.67± 3.88 hours. Statistical analysis of postoperative data suggested that mean ICU stay was 33.14±16.11 hours and hospital stay was 5.81±1.19 days. There was not a single operative mortality. However one patient who was operated by intra cardiac baffle with single patch technique developed juntional rhythum which was well controlled on amiodarone. One patient developed mild SVC obstruction having 6mmHg gradient and is doing well on follow up since 9 months.

## DISCUSSION

Various techniques have been described for correction of PAPVC and these repair techniques have evolved over years. For right sided PAPVC to SVC and sinus venosus ASD, patch baffling has been described between the right sided anomalous pulmonary veins and left atrium along with with an additional patch to augment SVC.^9^ Warden technique has also been suggested which involves SVC reimplantation into right atrial appendage along with patch baffling of SVC orifice.^10^,^11^ The idea is to eliminate the intra atrial communication without narrowing of pulmonary vein or SVC and avoiding any injury to sinus node.

**Table-II:** Per-operative findings, Length of ICU stay, Hospital stay and Operative mortality.

Bypass Time (min)	80.70±22.95
Cross-clamp Time (min)	45.46±15.63
Ventilation Time (Hours)	5.67±3.88
ICU Stay (Hours)	33.14±16.11
Hospital Stay (Days)	5.81±1.19
Operative Mortality (%)	0.0 (0.00)

Earlier baffle repair was associated with complications like SVC stenosis, pulmonary vein orifice obstruction, sinus node injury atrial bradyarrythmias, however recent case series suggest reassuring outcomes.^12^,^13^ Cavoatrial incisions through sinus node was associated with rhythum abnormalities.^14^

Therefore, appropriately planned incision can reduce chances of rhythum abnormalities. We used predominantly, intracardiac baffle with double patch technique in right-sided PAPVC to SVC with very low incidence of surgical morbidity. Though we employed intrcardiac baffle with single patch in few cases and warden technique in one case. We managed to avoid sinus node injury by carefully confining the incision at the SVC and right atrial interface to posterior and lateral area. Thus avoiding brady-arrhythmia and tachyarrhythmia. Only one of our patients developed SVC stenosis having 6mmHg gradient. Our results are highly comparable to international quoted statistics. Although number of cases are limited in our study but considering high success rate of corrective surgery along with low morbidity and no mortality it can be taken as positive step forward.

## CONCLUSION

PAPVC repair can be accomplished effectively with minimal morbidity. The double patch technique in right sided PAPVC carries highly reproducible outcome with greatly reduced morbidity in terms of SVC narrowing and pulmonary vein gradient.
